# Simple and rapid hydrogenation of *p*-nitrophenol with aqueous formic acid in catalytic flow reactors

**DOI:** 10.3762/bjoc.9.129

**Published:** 2013-06-14

**Authors:** Rahat Javaid, Shin-ichiro Kawasaki, Akira Suzuki, Toshishige M Suzuki

**Affiliations:** 1 Research Center for Compact Chemical System, National Institute of Advanced Industrial Science and Technology, AIST, 4-2-1 Nigatake, Miyagino-ku, Sendai, Miyagi 983-8551, Japan

**Keywords:** catalytic tubular reactor, flow chemistry, formic acid, hydrogenation, *p*-aminophenol, *p*-nitrophenol

## Abstract

The inner surface of a metallic tube (i.d. 0.5 mm) was coated with a palladium (Pd)-based thin metallic layer by flow electroless plating. Simultaneous plating of Pd and silver (Ag) from their electroless-plating solution produced a mixed distributed bimetallic layer. Preferential acid leaching of Ag from the Pd–Ag layer produced a porous Pd surface. Hydrogenation of *p*-nitrophenol was examined in the presence of formic acid simply by passing the reaction solution through the catalytic tubular reactors. *p-*Aminophenol was the sole product of hydrogenation. No side reaction occurred. Reaction conversion with respect to *p*-nitrophenol was dependent on the catalyst layer type, the temperature, pH, amount of formic acid, and the residence time. A porous and oxidized Pd (PdO) surface gave the best reaction conversion among the catalytic reactors examined. *p*-Nitrophenol was converted quantitatively to *p*-aminophenol within 15 s of residence time in the porous PdO reactor at 40 °C. Evolution of carbon dioxide (CO_2_) was observed during the reaction, although hydrogen (H_2_) was not found in the gas phase. Dehydrogenation of formic acid did not occur to any practical degree in the absence of *p*-nitrophenol. Consequently, the nitro group was reduced via hydrogen transfer from formic acid to *p*-nitrophenol and not by hydrogen generated by dehydrogenation of formic acid.

## Introduction

The flow reaction process enables continuous material production simply by feeding the reactants into one end of the reactor and obtaining the products from the other end [1–9]. Recently, we developed catalytic tubular reactors of less than 0.5 mm inner diameter, of which the interior surfaces were coated uniformly with thin (1–2 μm) palladium (Pd), platinum (Pt), and rhodium (Rh) layers by an electroless plating procedure [10–11]. These tubular reactors combined the merit of flow reaction processing with the catalytic properties of various metals. Unlike packed-bed catalysts, hollow tubular reactors can minimize the mass transfer resistance and therefore ensure a smooth flow of reactants without causing an undesirable pressure drop or clogging of reactor tubes. In addition, the tubular reactor offers a large surface-to-volume ratio, good mixing and heat-transfer properties that enhance the reaction rate [5]. We have studied flow reactions, including the decomposition of hydrogen peroxide, oxidation of organic dyes, carbon–carbon coupling, and conversion of formic acid to hydrogen (H_2_) and carbon dioxide (CO_2_), using catalytic tubular reactors [10–13].

*p*-Aminophenol is an important intermediate produced in the syntheses of various chemicals including dyes, pharmaceuticals, and anticorrosive lubricants [14–16]. The catalytic hydrogenation of aromatic nitro compounds with H_2_ has been studied extensively in the presence of Pd, Pt, Ni, and Rh metals [14,16–22]. In the light of the commercial importance of *p*-aminophenol, improvement of catalytic performance was attempted by using nanoparticles (NPs) of Au, Pd, and Ni immobilized on various solid supports. Apart from the use of gaseous H_2_, the reduction of *p*-nitrophenol with sodium borohydride (NaBH_4_) was studied catalyzed with core–shell Au–Pd NPs and Au NPs [23–26]. Formic acid is another attractive H_2_ source because it is safe, easy to handle, and requires no high-pressure equipment. Formic acid and formate have been used as effective H_2_ donors in the catalytic transfer hydrogenation of aromatic nitro compounds [27–31].

Here we attempted the hydrogenation of *p*-nitrophenol with formic acid in catalytic flow-through tubular reactors. Because Pd-based catalysts have usually been regarded as the most active catalysts [27–28,32], we modified the inner surface of tubular reactors with thin Pd, porous Pd, and their oxidized metal layers. Herein, we present a simple, rapid and convenient process for the reduction of *p*-nitrophenol, which is compatible with high reaction conversion under mild conditions.

## Results and Discussion

### Fabrication of the catalytic tubular reactors

**Figure 1:**
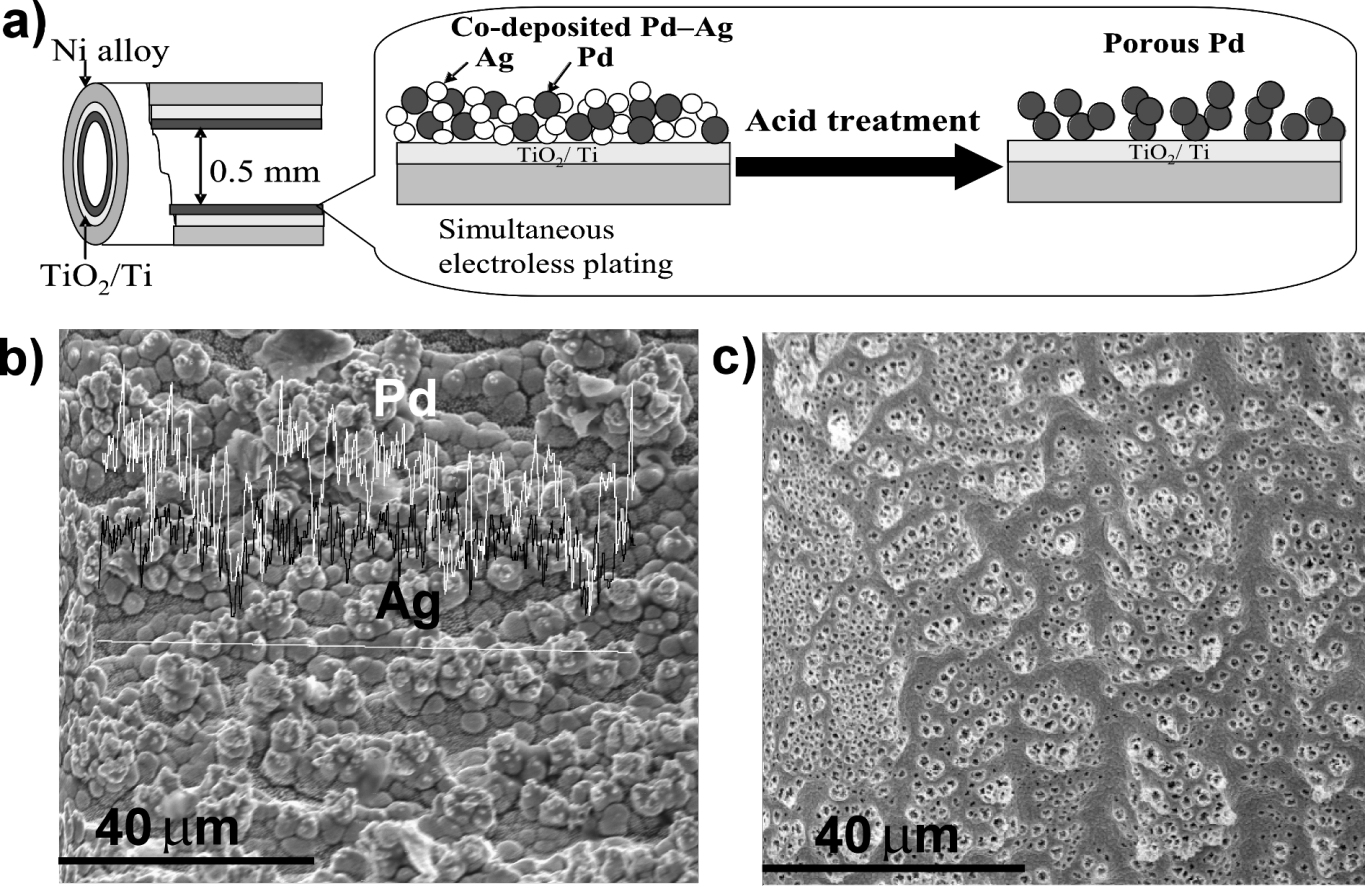
**(**a) Graphical presentation of Pd–Ag co-plating and sequential removal of Ag to give a porous Pd surface. (b) SEM image of Pd–Ag co-plated surface along with EDX presentation of Pd and Ag deposition. (c) Surface morphology of porous Pd.

Electroless plating is a simple and efficient methodology to coat the inner wall of a tubular reactor with various thin metal layers. Aside from the plating of a single Pd layer, we examined co-plating of Pd and Ag from their 9:1 (atomic ratio) mixed solution, as depicted in Figure 1a. Metal ions in the plating solution are stabilized against precipitation by complex formation with EDTA and NH_3_, which also controls the deposition rate of the individual metal by modifying the redox potential [33]. SEM and EDX analysis of the plated layer indicated the mixed distribution of Pd and Ag over the inner surface (Figure 1b). It has been observed that Ag is preferentially plated. Therefore, a small excess of Ag (13%) was deposited over the atomic ratio present in the plating solution [33]. When the Ag content exceeds 15% in the Pd–Ag co-plating, the plating solution becomes considerably unstable and tends to give undesirable precipitation.

Selective dissolution of the less-noble element out of a bimetallic mixture or alloy results in the formation of a unique metallic sponge structure of the noble component [34–35]. We attempted to modify the reactor wall with a porous Pd layer. Continuous passage of 4 M HNO_3_ into the reactor with a Pd–Ag co-plated layer preferentially dissolved Ag, leaving pores behind. Figure 1c shows an SEM image of the porous Pd surface after removal of Ag, where numerous pores are observed. Direct determination of the porosity of the porous metal layer was difficult because it firmly adhered and resisted removal from the inner surface of the narrow tube. Instead we conducted similar co-plating of Pd and Ag (9:1) on a glass surface. The plated Pd–Ag film was peeled from the glass surface. Then Ag leached out by acid treatment of the film. The BET specific surface area and average pore diameter were determined respectively as 8.8 m^2^ g^−1^ (106 m^2^ cm^−3^ as for volume base) and 9.4 nm.

The oxidized palladium (PdO) surface often gave high catalytic activity [11,13,36–41]. Air oxidation of Pd and the porous Pd layer in the tubular reactors was conducted at 750 °C under air flow for 2 h. The XPS analysis confirmed complete oxidation of the Pd surface to PdO, as presented in our previous study [13].

### Hydrogenation of *p*-nitrophenol in the catalytic flow reactors

**Figure 2:**
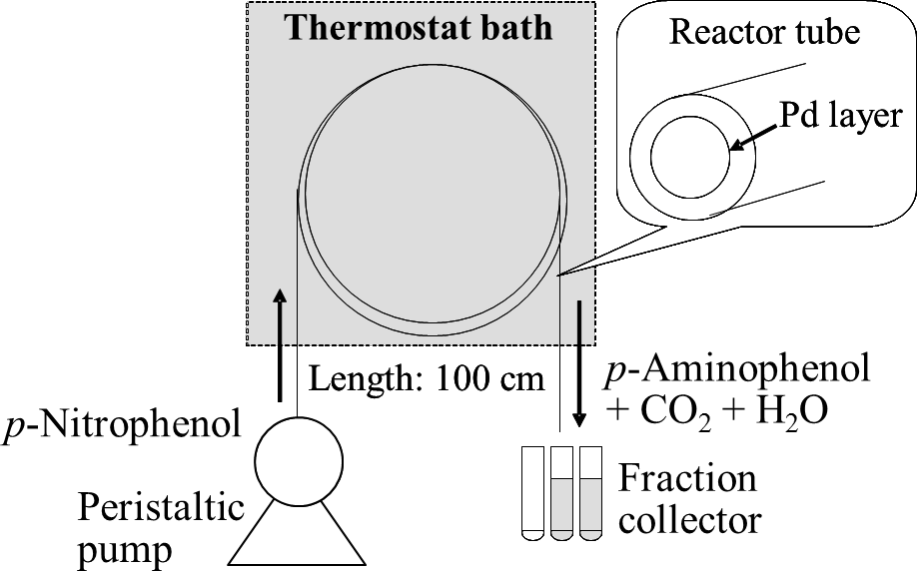
Schematic diagram of experimental setup used for the catalytic hydrogenation of *p*-nitrophenol.

The experimental setup of our flow reaction system is simple, as depicted in Figure 2, where a reactor tube loop was immersed in a water bath maintained at constant temperature. An aqueous solution containing *p*-nitrophenol and formic acid was supplied continuously from one end and collected in fractions at the other open end. The reaction solution flow is smooth and stable because the catalytic reactors are hollow tubes, avoiding the pressure drop and plugging.

**Scheme 1:**
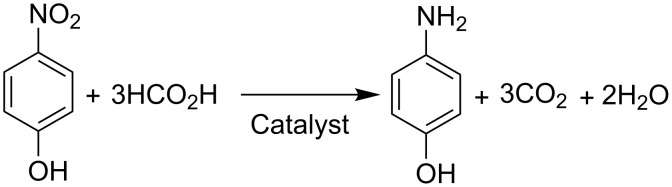
Hydrogenation of *p*-nitrophenol with formic acid.

The evolution of gas bubbles was observed during the reaction forming an alternate gas–liquid slug flow. GC analysis of the gas phase evidenced the evolution of CO_2_ as a sole product and H_2_ was not found. The hydrogenation reaction of *p*-nitrophenol was followed by UV–vis spectroscopy. The analytical UV–vis peaks of *p*-nitrophenol and *p*-aminophenol are sufficiently separated both under acidic and basic conditions (Supporting Information File 1). The spectral difference between acidic and basic solutions is responsible for the association and dissociation of phenolic proton. The concentration of *p*-nitrophenol was determined by absorbance at 317 nm (acidic conditions) using the calibration curve. The presence of isosbestic points in the spectra (Supporting Information File 1) of the reaction mixture indicates that *p*-aminophenol is a sole product in the solution, and consequently no side reaction occurs as expressed in Scheme 1.

### Catalytic activity of the reactors

**Figure 3:**
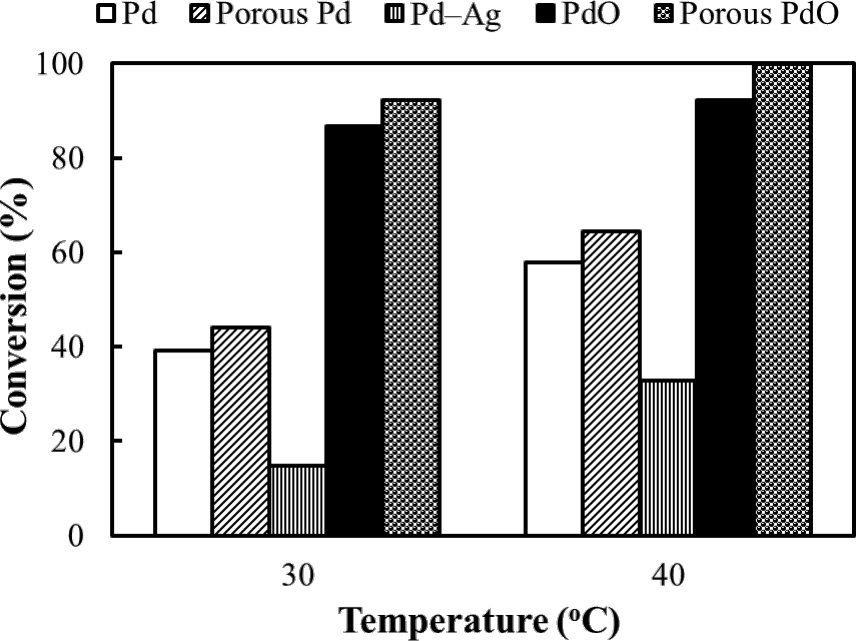
Influence of temperature on the conversion of 0.01 M *p*-nitrophenol with 0.1 M formic acid at 30 °C and 40 °C. Tubular reactors coated with Pd, PdO, Pd–Ag, porous Pd or porous PdO were applied for demonstration of the catalytic activity. All experiments were conducted with 14.7 s residence time.

Hydrogenation of 0.01 M *p*-nitrophenol with 0.1 M formic acid was conducted in aqueous solution at 30 °C and 40 °C by using tubular reactors coated with Pd, porous Pd, metallic Pd–Ag and porous PdO. The pH of the aqueous reaction solution as prepared was 2.2. The flow rate was fixed to 0.8 mL min^−1^, which corresponds to 14.7 s of residence time in the tubular reactor. Figure 3 shows the reaction conversion of *p*-nitrophenol obtained by using the respective tubular reactors. The catalytic surface unquestionably contributed to facilitation of the reaction, because practically no reaction took place in the absence of the catalytic layer, even at 70 °C. It is noteworthy that oxidation of the Pd surface improved the catalytic activity remarkably. The number of surface hydroxy groups increases with the oxidation of Pd, allowing more intimate interaction with the reactants (see below). Addition of 13% Ag to Pd greatly decreased the catalytic activity, presumably because Ag suppresses and inhibits the active Pd site. In fact, when Ag was removed by acid, the catalytic activity was revived. Moreover, the reactor with a porous PdO surface gave markedly superior conversion (>99 %) compared to that of the corresponding nonporous counterparts.

**Figure 4:**
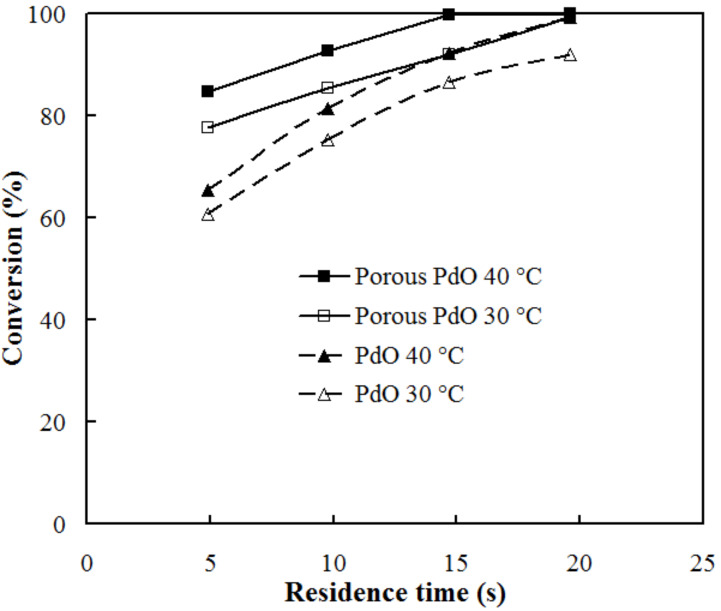
Effect of residence time on the conversion of 0.01 M *p*-nitrophenol with 0.1 M formic acid at 30 °C and 40 °C using PdO and porous-PdO-coated tubular reactors.

Superior catalytic activity of the porous surface was also confirmed by the effect of the residence time (flow rate) in the reactors. As presented in Figure 4, the reactor of porous PdO invariably attained higher conversion with shorter residence time than any other reactor. For example, 100% conversion was attained after 14.7 s residence time for porous PdO, whereas nonporous PdO required 19.6 s. The porous and rough reactor surface provided more contact opportunity with substrates, with which a much higher surface-area-to-volume ratio can be attained. The total amount of Pd present in the tube inner layer does not represent the concentration of active catalyst, since only surface metal atoms work as active sites. Therefore we estimated the number of surface Pd atoms using the observed surface area of porous Pd, the number of closely packed Pd atoms in a face centred cubic (fcc) crystal unit, and the atomic radius of Pd (0.137 nm) [32]. The catalytic activity expressed in terms of turnover frequency (TOF) was calculated to be around 320 h^−1^ (mol of substrate per mol of Pd site per hour) at 40 °C. This value is much higher than those reported for the catalytic transfer hydrogenation of *p*-nitrophenol and benzyl acetate in a flow system [30,32].

**Figure 5:**
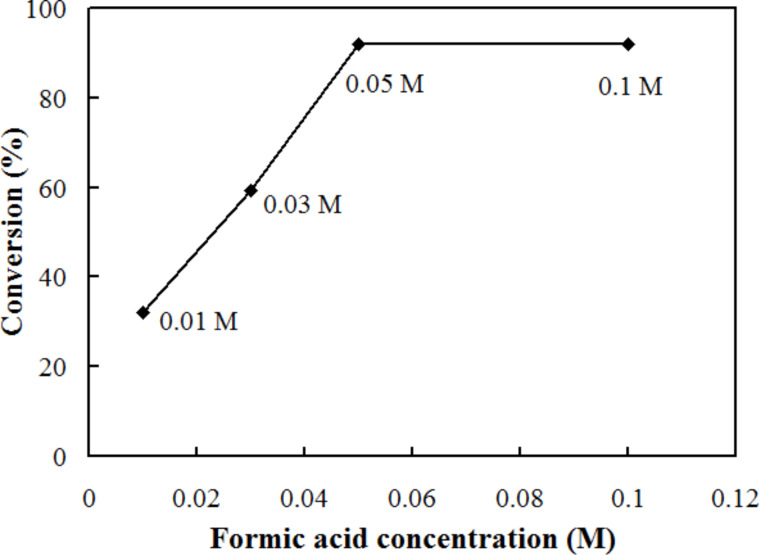
Effect of the concentration of formic acid on the conversion of 0.01 M *p*-nitrophenol. The formic acid concentration varied: 0.01 M, 0.03 M, 0.05 M and 0.1 M. Porous-PdO-coated reactor was used at 30 °C, with a residence time of 14.7 s.

We conducted the reaction while changing the concentration of formic acid and maintaining the *p*-nitrophenol concentration to 0.01 M. According to the reaction stoichiometry expressed in Scheme 1, three times the molar concentration of formic acid is necessary for the reduction of one mole of *p*-nitrophenol. In the present flow reaction, at least 0.05 M formic acid was necessary to attain the maximum reaction conversion of 92.1% at 30 °C (Figure 5), which corresponds to a 1.7 times excess of the reducing agent to the *p*-nitrophenol. Further increases of concentration did not improve the reaction outcomes (Figure 5).

### Transfer hydrogen from formic acid to *p*-nitrophenol

Dehydrogenation of formic acid and its subsequent reduction of nitro group is a possible reaction pathway. However as mentioned, CO_2_ was a sole product in the gas phase and H_2_ was not found even at an increased temperature of 70 °C. To ensure that the nitro group is not reduced by H_2_ generated by dehydrogenation of formic acid, we conducted a control test where *p*-nitrophenol was absent. Notably when *p*-nitrophenol was not present in the reaction solution formic acid was not consumed at all at the present reaction temperature. In our previous study, more than 250 °C was necessary for the dehydrogenation of formic acid in the Pd-coated tubular reactor [13]. Consequently, we concluded that the nitro group was reduced in a transfer hydrogenation process and not by molecular H_2_ generated from the dehydrogenation of formic acid. In contrast to hydrogenation using gaseous H_2_, the present system using formic acid as hydrogen donor has the advantages of convenience and safety.

**Figure 6:**
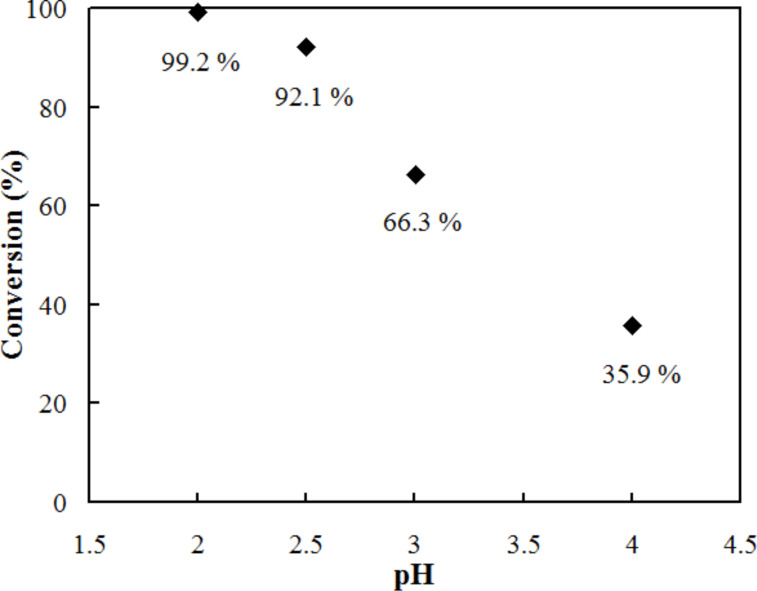
Effect of pH on the conversion of 0.01 M *p*-nitrophenol by using 0.05 M formic acid. The porous PdO coated reactor was used at 30 °C and a residence time of 14.7 s.

The PdO surface has a higher number of hydroxy groups than that of metallic Pd providing more opportunity for interaction sites with the substrates. This is in accordance with the higher reaction conversion attained by oxidized PdO reactor tube than that by the metallic Pd reactor tube. The reaction efficiency in a porous PdO reactor apparently depends on the pH of the reaction solution, as given in Figure 6. The decrease of pH produced markedly better conversion. By considering the p*K*_a_ of formic acid (3.5), the acid form (HCO_2_H) contributes to the reaction conversion better than basic formate (HCO_2_^−^) does. H_2_ transfer from formic acid to a nitro compound has often been facilitated in the presence of a base, such as triethylamine and NH_3_ [28,32,42–44]. Therefore, the addition of such bases is usually necessary for transfer hydrogenation. As in the present case, base-free catalytic transfer hydrogenation of a nitro compound is a rather rare example [31,45]. One plausible explanation is a pH dependence of the electrostatic interaction between formic acid/formate and the catalytic surface. The oxidized Pd surface (PdO) is equilibrated with Pd–OH and Pd–O^−^ in aqueous solution depending on the pH [46]. Increase in the number of Pd–O^–^ at high-pH region makes the surface more negative and inhibits the access of formate (HCO_2_^−^) and phenolate (NO_2_–Ph–O^−^) anions, although at low pH, such repulsion of Pd–OH and the acidic reactants is suppressed.

### Long-term stability

**Figure 7:**
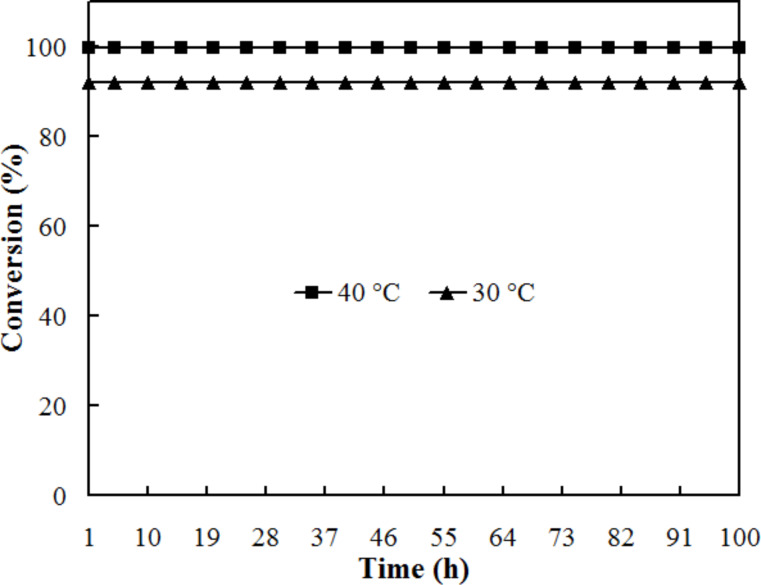
Long-term testing for continuous hydrogenation of 0.01 M *p*-nitrophenol with 0.05 M formic acid in the porous PdO-coated reactor. Residence time: 14.7 s.

To evaluate the long-term stability and activity of the catalytic reactor, the reaction was run for 100 h at 30 °C and another 100 h at 40 °C. In these experiments, a solution of 0.01 M *p*-nitrophenol and 0.05 M formic acid was fed continuously to the reactor tube at a fixed residence time of 14.7 s. The solution was collected out of the reactor in fractions and the conversion was determined. As Figure 7 shows, the reaction was stable over this period. Palladium was not found in the reaction solution by the ICP–AES analysis, indicating that the leaching of Pd from the reactor tube was negligible during these experimental runs. Unlike other catalytic approaches for the reduction of *p*-nitrophenol [25,47–49], our catalytic flow system requires no product separation procedure. Moreover, we observed no decrease in the catalytic activity.

## Conclusion

A flow electroless plating procedure was employed to produce a thin metal-catalyst coating layer over the inner surface of a tubular reactor. Co-plating of Pd and Ag yielded mixed distributed Pd and Ag inner surfaces, which further gave a porous Pd surface by preferential dissolution of Ag by HNO_3_. A hollow tubular reactor combined with the catalytic inner surface enabled rapid and continuous reaction under a smooth flow of reactants and products. The versatility of these catalytic tubular reactors was demonstrated through efficient and rapid reduction of *p*-nitrophenol by using formic acid. The nitro group is reduced in a transfer hydrogenation process and not by molecular H_2_ generated from dehydrogenation of formic acid. The porous and oxidized Pd surface showed better catalytic activity because of the increased surface area and roughness. No significant sign of deactivation of the catalyst or leaching of Pd was observed through 100 h continuous reaction, which demonstrates the robustness of the present catalytic reactors.

## Experimental

### Materials and reagents

Reagent-grade formic acid (HCO_2_H, 98%), *p*-nitrophenol, palladium acetate [Pd(CH_3_COO)_2_], silver nitrate (AgNO_3_), disodium ethylenediamine tetraacetate (EDTA–Na_2_), ammonia (NH_3_, 28%), hydrazine monohydrate, nitric acid (HNO_3_, 60%) and hydrogen peroxide (H_2_O_2_, 30%) were purchased from Wako Pure Chemical Industries Ltd. and were used without further purification.

#### Fabrication of tubular reactors

A double-layered tube (o.d. 1.6 mm, i.d. 0.5 mm, length 100 cm) composed of Inconel 625 and titanium (Ti) inner layer (thickness 120 μm) was fabricated by E.S.Q. Co., Japan, by elongation of the titanium inlaid Inconel 625 piece by stretch-draw process, and used as the reactor support [11]. The Ti inner surface was oxidized to TiO_2_ with H_2_O_2_ under supercritical water conditions (450 °C, 30 MPa). The TiO_2_ surface, which is suitable for Pd plating, also acts as a barrier to prevent intermetallic diffusion of Ti and the metal catalyst. The TiO_2_ surface was activated with Pd seeds before electroless plating. Plating of Pd was then conducted according to the procedure described in previous reports [10–12]. By 5 h plating at 50 °C, 67.3 mg of Pd was deposited, which corresponds to a Pd inner wall thickness of 3.6 μm. The Pd surface was oxidized by calcination of the tubular reactor at 750 °C for 2 h under air flow.

Co-plating of Pd and silver (Ag) was typically performed by passing (0.5 mL min^−1^) an aqueous solution containing 9 mM Pd(CH_3_COO)_2_, 1 mM AgNO_3_, 0.15 M EDTA–Na_2_, 4 M NH_3_, and 10 mM hydrazine monohydrate through the reactor tube at 60 °C. After 3 h plating, 67.7 mg of Pd (87%) and 10.1 mg of Ag (13%) were deposited inside the tubular reactor. After plating, washing with water was conducted to remove the chemicals remaining inside the tube. The Pd–Ag mixed layer thickness was estimated as ca. 4.2 μm.

Subsequent removal of Ag by passing 4 M HNO_3_ (840 mL) at 25 °C with a flow rate of 0.5 mL min^−1^ gave the porous Pd surface as observed by SEM imaging. The remaining amounts of Pd and Ag were 48.1 mg (94%) and 2.9 mg (5.7%), respectively. We separately co-plated Pd and Ag film on a glass surface with the same chemical composition. Then the thin film (3.5 μm) was peeled and treated with 4 M HNO_3_ and provided to the BET specific surface area and pore diameter measurements at 77 K by nitrogen absorption isotherm.

#### Hydrogenation of *p*-nitrophenol

A typical reaction procedure is the following, using the experimental setup depicted in Figure 2. The reactor tube and container of reaction stock solution were immersed in a water bath to maintain a constant temperature. The reaction was conducted at ambient pressure. An aqueous solution containing *p*-nitrophenol (10 mM) and formic acid was fed into the tubular reactor (100 cm long with inner volume of 196 μL) at a constant flow rate controlled by a peristaltic pump. The solution out of the reactor tube was collected in fractions and the UV–vis absorption spectra were measured. The analytical UV–vis peaks of substrate and product are sufficiently separated and the concentration of *p*-nitrophenol was ascertained by the absorbance at 317 nm using the calibration curve. The residence time was estimated from the volume of reaction solution at a fixed time divided by the inner volume of the reactor tube. Leaching of Pd from the reactor tube during the reaction was checked by inductively coupled plasma–atomic emission spectroscopy (ICP–AES) analysis of the fractionated reaction solution.

#### Instruments

The metal concentration was analyzed using ICP– atomic emission spectroscopy (ICP–AES, Model SPS 3100; SII Nano Technology Inc.). The morphology of the inner surface of the catalytic tubular reactors was observed by scanning electron microscopy (SEM) equipped with an energy-dispersive X-ray spectrometer (EDX, XL30S; Philips Co.). UV–vis absorption spectra were recorded at room temperature by using a spectrophotometer (U–3310; Hitachi). The collected gaseous products were analyzed by using a gas chromatograph (GC–8A; Shimadzu Corp.) equipped with thermal conductivity detector (TCD). A molecular sieve (5 Å) column (3 mm × 6 m) was used for H_2_ with argon as the carrier gas, whereas a Porapak–Q column (3 mm × 3 m) was used coupled with helium as the carrier gas for CO_2_ analysis. The specific surface area and pore diameter of the porous Pd were determined volumetrically (N_2_ adsorption) by using Belsorp MAX. Samples were degassed at 250 °C under vacuum prior to the measurement.

## Supporting Information

The Supporting Information features the isosbestic points in the UV–vis spectra of the reaction mixture indicating that *p*-aminophenol is the sole product and no side reaction occurs.

File 1 Analytical and spectral data.
