# Supplementary material for: Simple and rapid hydrogenation of p-nitrophenol with aqueous formic acid in catalytic flow reactors
Source: Beilstein J Org Chem. 2013 Jun 14;9:1156–63. doi: 10.3762/bjoc.9.129 (PMC3701373; doi:10.3762/bjoc.9.129)
Supplement: File 1 — Analytical and spectral data. [file Beilstein_J_Org_Chem-09-1156-s001.pdf]

Supporting Information

for

Simple and rapid hydrogenation of *p*-nitrophenol with aqueous formic acid in catalytic flow reactors

Rahat Javaid*, Shin-ichiro Kawasaki, Akira Suzuki and Toshishige M. Suzuki*

Address: Research Center for Compact Chemical System, National Institute of Advanced Industrial Science and Technology, AIST, 4-2-1 Nigatake, Miyagino-ku, Sendai, Miyagi 983-8551, Japan

Email: Rahat Javaid* - rahat-javaid@aist.go.jp; Toshishige M. Suzuki* - tm-suzuki@aist.go.jp

* Corresponding author

Analytical and spectral data

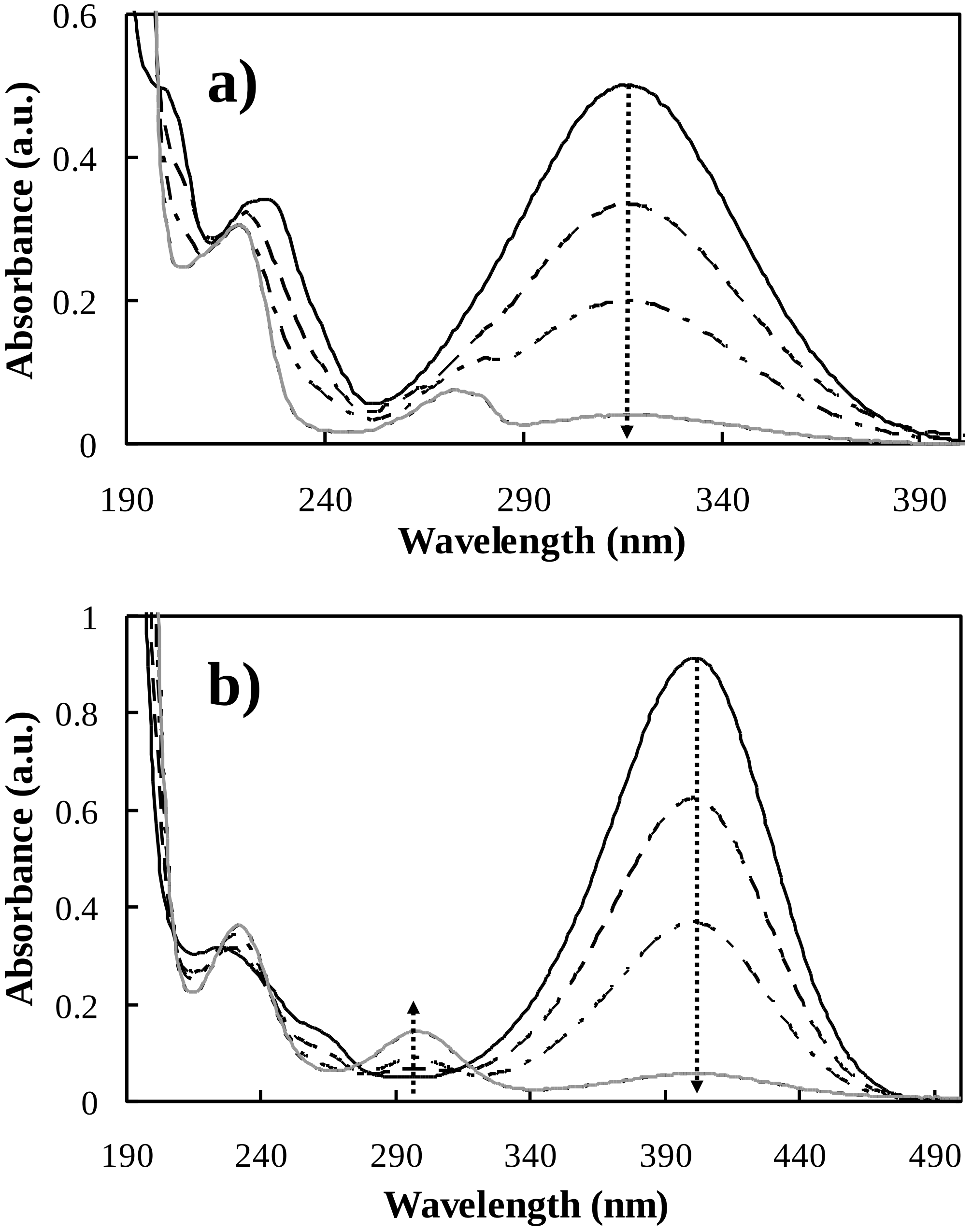

Figure: UV–vis spectral change of *p*-nitrophenol during the hydrogenation reaction by use of different concentrations of formic acid (——— Original – – – 0.01 M – · – 0.03 M ········· 0.05 M ——— 0.1 M). Measured pH (a) pH = 2.5 and (b) pH = 5.0. Concentration of *p*-nitrophenol = 1×10^{-4} M.
